# Toxic Shock Syndrome: A Rare but Dangerous Adverse Effect of Anabolic Steroid Injection

**DOI:** 10.1002/ccr3.70789

**Published:** 2025-08-15

**Authors:** Elise Van der Borght, Thomas Demuynck, Lauren Keuleers, Marijke Peetermans, Peter Vanbrabant

**Affiliations:** ^1^ Department of General Internal Medicine University Hospitals Leuven Leuven Belgium; ^2^ Department of General Internal Medicine, Department of Medical Intensive Care University Hospitals Leuven Leuven Belgium; ^3^ Department of Radiology University Hospitals Leuven Leuven Belgium; ^4^ Medical Intensive Care Unit, Department of General Internal Medicine University Hospitals Leuven Leuven Belgium; ^5^ Laboratory for Clinical Infectious and Inflammatory Disorders, Department of Microbiology, Immunology and Transplantation KU Leuven Leuven Belgium

**Keywords:** emergency medicine, infectious diseases, radiology and imaging, surgery

## Abstract

This case highlights the risk of staphylococcal TSS following an anabolic steroid injection‐related thigh infection. Prompt recognition and treatment are crucial for preventing rapid deterioration. It also underscores the importance of thorough source review, infection control, and awareness of the risks associated with anabolic steroid use.

## Graphical abstract

Toxic shock syndrome (TSS) is a life‐threatening, toxin‐mediated disease characterized by hypotension, fever, diffuse erythema, and multi‐organ failure. Early recognition and adequate treatment are paramount. The main causative agents are Streptococcus pyogenes and Staphylococcus aureus. TSS can be associated with focal infections such as soft tissue infections and postsurgical wound infections, as well as with burns, local injuries or postpartum and postabortion infections. We present the unique case of a 39‐year‐old male personal trainer who presented to the emergency department with a staphylococcal toxic shock syndrome secondary to an anabolic steroid injection‐related surinfected collection of the thigh. This case report demonstrates the importance of rapid recognition and treatment of toxic shock syndrome. It highlights the importance of a thorough source review and control and discusses the risks of using anabolic steroid injection.

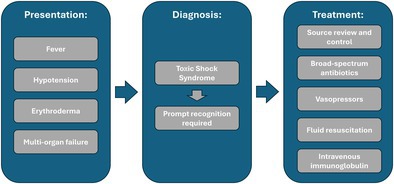

## 1 Introduction

Toxic shock syndrome (TSS) is a critical, toxin‐mediated disease characterized by hypotension, fever, diffuse erythema, and multi‐organ failure [1, 2, 3, 4, 5, 6]. If not recognized rapidly and treated correctly, it is potentially lethal. *Streptococcus pyogenes* and Panton‐Valentine Leucocidin‐producing *Staphylococcus aureus* are the predominant causative bacteria of TSS [1, 2, 3, 4, 5, 6]. The annual incidence is 1.5–11 per 100,000 people and occurs most often at the extremes of ages [1]. Sources of nonmenstrual staphylococcal TSS can be postsurgical wounds, postpartum infection, soft tissue injuries, and necrotizing pneumonia [1, 5]. For streptococcal TSS, soft tissue infections, including necrotizing fasciitis, and focal portals of entry, such as pneumonia and ear/nose/throat infections, have been described [1].

We present the unique case of a 39‐year‐old male personal trainer who presented to the emergency department with a flu‐like picture of erythroderma and rapidly progressed to multi‐organ failure.

## 2 Case History/Examination

A 39‐year‐old personal trainer with a past medical history of Hashimoto thyroiditis and body dysmorphic disorder presented to our emergency department with myalgia and progressive weakness, anorexia, and malaise. His regular medication consisted of levothyroxine, atypical antipsychotics, antidepressants, benzodiazepines, and phosphodiesterase type 5 inhibitors.

**FIGURE 1:**
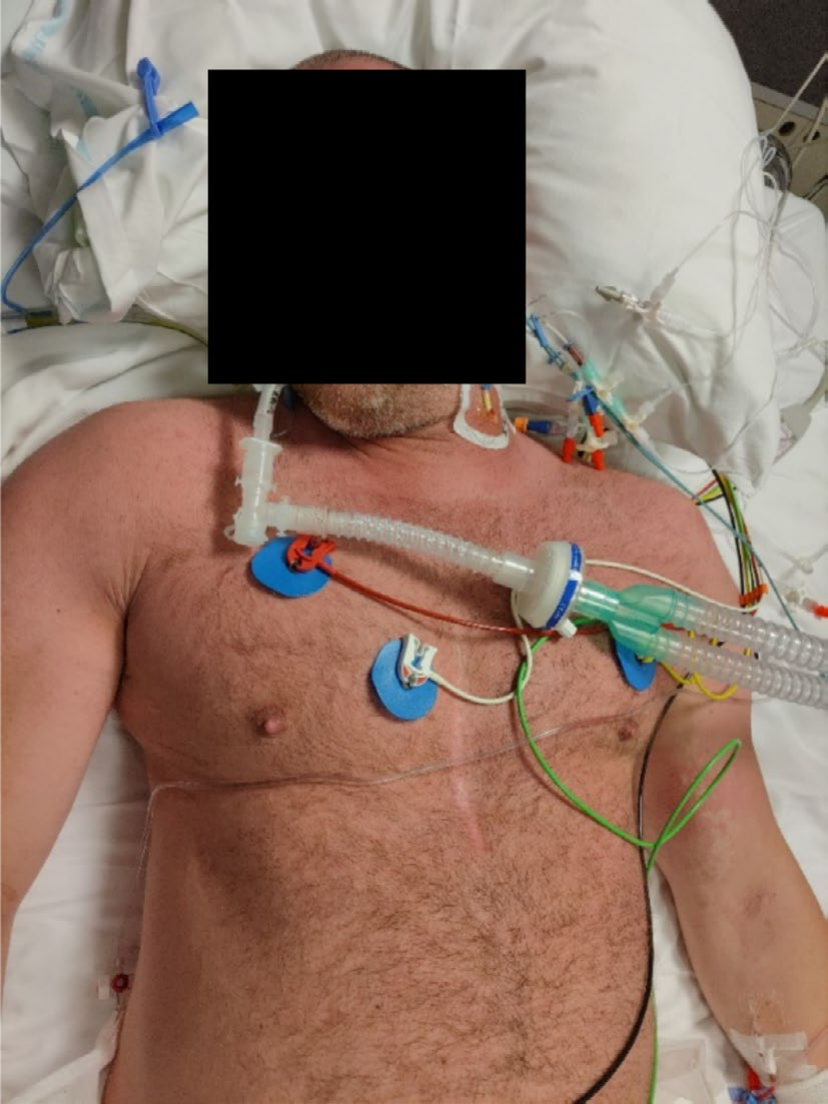
Clinical photograph revealing diffuse erythroderma to the entire face/neck/upper chest.

Upon arrival at the emergency department, his initial vital signs revealed tachycardia at 120 bpm, a temperature of 38.4°C, blood pressure of 95/51 mmHg, and pulse oximetry of 95% on room air. On general examination, the patient had diffuse erythema (Figure 1). He appeared in respiratory distress with tachypnea. On lung auscultation, we heard decreased breath sounds bilaterally. His remaining physical examination was unremarkable.

## 3 Differential Diagnosis, Investigations, and Treatment

### 3.1 Differential Diagnosis

The patient's presentation with fever, hypotension, erythroderma, and multi‐organ failure raised suspicion for several conditions, including [2].

- Scarlet fever
- Meningococcemia
- Toxic epidermal necrolysis
- Hemorrhagic shock
- Necrotizing Fasciitis/Gas gangrene
- Toxic shock syndrome
- Drug Reaction with Eosinophilia and Systemic Symptoms (DRESS)
- Anaphylactic shock

### 3.2 Investigations

The initial laboratory results were remarkable for leucocytosis and an elevated C‐reactive protein of 306.8 mg/L. In addition, this also revealed acute kidney injury stage III, with a serum creatinine at presentation of 8.76 mg/dL, a normal baseline serum creatinine of 0.94 mg/dL, and elevated creatine kinase of 346 U/L.

The chest x‐ray showed bilateral pulmonary opacities, without pleural effusion and an enlarged cardiac silhouette.

**FIGURE 2:**
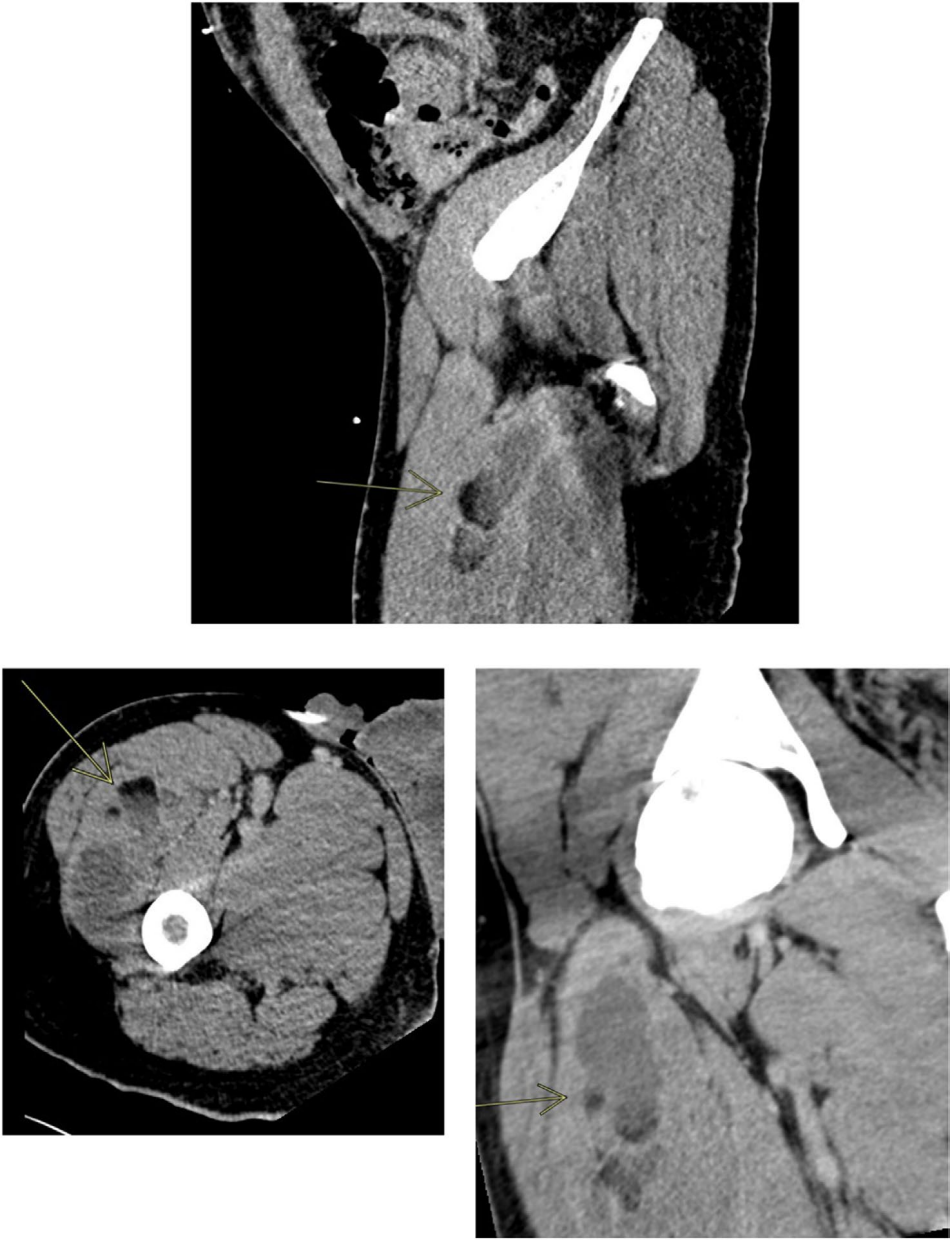
CT scan demonstration incompletely depicted large intramuscular collection in the right vastus intermedius muscle.

Given the suspicion of TSS and the absence of a clinically obvious source, a computed tomography (CT) of the chest and pelvis was performed. The CT scan incidentally demonstrated a multilocular collection (6.8 × 3.2 × 311 cm) in the right vastus intermedius muscle. This collection had a fat‐fluid level, mild peripheral contrast enhancement, and streaky infiltration of the left vastus intermedius muscle (Figure 2).

### 3.3 Treatment

The patient was empirically started on intravenous antibiotics (ceftriaxone, clindamycin, and flucloxacillin) and fluid resuscitation. He also received intravenous immunoglobulins (IVIG) at a dose of 1 g/kg awaiting cultures. In the next hours, however, he rapidly became hypotensive and hypoxic and required endotracheal intubation and mechanical ventilation. Further hemodynamic deterioration led to the addition of norepinephrine, vasopressin, and hydrocortisone to his therapy. An urgent bedside echocardiography was suggestive of septic cardiomyopathy with a reduced left ventricular ejection fraction of 40%.

Considering the clinical presentation and imaging, TSS secondary to an anabolic androgenic steroid (AAS) injection‐related surinfected collection of the thigh was suspected.

He underwent emergency drainage with debridement. Blood cultures remained negative, but collection fluid grew methicillin‐sensitive *Staphylococcus aureus* . Clindamycin was stopped after a total duration of 3 days after the resolution of shock. Flucloxacillin monotherapy was continued. Given the development of cholestasis secondary to treatment with flucloxacillin, this was replaced by clindamycin. Antibiotic treatment was administered for a total of 14 days. Under the established management plan, a favorable progression was observed, with a gradual reduction in vasopressin requirements.

## 4 Conclusion and Results

This case highlights a rare presentation of staphylococcal TSS secondary to an AAS injection‐related infection. Given the potential for rapid clinical deterioration, prompt recognition and targeted treatment were essential. Additionally, it emphasizes the importance of a thorough source review and control, and highlights the risks associated with using AAS injections. The patient showed significant clinical improvement following appropriate antimicrobial therapy and surgical debridement. He was successfully weaned off vasopressors and extubated, with a full recovery at follow‐up.

## 5 Discussion

The case definition for staphylococcal TSS from The Centers for Disease Control and Prevention is based on clinical and laboratory criteria [1, 5]. The clinical criteria included a fever above 38.9°C or 102.0°F, rash with diffuse macular erythroderma with desquamation 1–2 weeks after rash onset, hypotension with SBP ≤ 90 mmHg for adults, and multi‐organ involvement (three or more systems). Blood cultures can be negative or grow *Staphylococcus aureus* [1, 5]. This definition has several limitations as it was designed for study purposes (high specificity) [4]. Some criteria cannot be observed at presentation, such as desquamation that only develops 8 to 21 days after the onset of the disease [4, 6]. In this case, the clinical team's early recognition based on case definition—fever, rapid hemodynamic deterioration, multi‐organ involvement, and erythroderma—was essential to initiate rapid and targeted therapy. Following the latest guidelines, we treated the patient with fluid resuscitation, broad‐spectrum antibiotics, vasopressors, and source control by debridement [1, 2, 4, 5, 6]. Awaiting the results of the cultures, IVIG and clindamycin were added to the therapy. However, there is a lack of robust evidence of IVIG and clindamycin in staphylococcal TSS [3, 4], while evidence in streptococcal TSS is stronger [7]. The use of adjunctive clindamycin, an antibiotic directed against exotoxin production in *Staphylococcus aureus* , will be investigated in a platform trial [8]. Locating the source of infection is important in managing sepsis in general and TSS. Extended imaging may be necessary if no clinically obvious source is found [4]. In this case, the patient showed no visible soft tissue injury. However, an intramuscular collection was observed on a CT scan. Based on the radiological findings, an intramuscular injection of anabolic steroids complicated by a surinfected collection formation was suspected. The patient later disclosed the use of AAS injections.

AAS are widely used, and the global lifetime prevalence rate is estimated at 6.4% for males and 1.6% for females [9]. AAS are abused for their muscle‐building properties. The well‐known side effects range from acne vulgaris, gynecomastia, and infertility to potentially life‐threatening ones such as erythrocytosis, hypertension, hepatotoxicity, nephrotoxicity, and cardiomyopathy [8]. The following risks are associated with the intramuscular use of AAS: local injuries and infections (such as abscesses, open wounds, and cellulitis) and systemic infections (such as hepatitis B, hepatitis C, and HIV) [10]. The most effective way to prevent these side effects is by discontinuing AAS use. If patients are reluctant to stop injections, education on sterile precautions is essential [10].

## Author Contributions

**Elise Van der Borght:** conceptualization, data curation, methodology, writing – original draft, writing – review and editing. **Thomas Demuynck:** writing – review and editing. **Lauren Keuleers:** writing – review and editing. **Marijke Peetermans:** supervision, writing – review and editing. **Peter Vanbrabant:** conceptualization, supervision, writing – review and editing.

## Consent

Written informed consent has been obtained.

## Conflicts of Interest

The authors declare no conflicts of interest.

## Data Availability

The data that support the findings of this case are available from the corresponding author upon reasonable request, subject to ethical and privacy restrictions.
